# Concomitant Percutaneous Coronary Intervention and Mitral Transcatheter Edge-to-Edge Repair for Acute Ischemic Mitral Regurgitation From Papillary Muscle Rupture

**DOI:** 10.1016/j.shj.2025.100723

**Published:** 2025-08-12

**Authors:** Luai Madanat, Rohit Chandra, Samia Mazumder, Richard Bloomingdale, Ahmad Jabri, Vishal Birk, Brian Renard, Rohit Vyas, Marina Maraskine, Ivan D. Hanson, Amr E. Abbas

**Affiliations:** Division of Cardiovascular Medicine, William Beaumont University Hospital, Royal Oak, Michigan, USA

**Keywords:** Acute ischemic MR, mitral transcatheter edge to edge repair, Papillary muscle rupture

## Abstract

• In high-risk patients, M-TEER is a feasible alternative to surgery for acute ischemic MR.
• In acute ischemic MR, M-TEER is challenged by limited atrial size, flail gaps, muscle impingement risk, and hemodynamic instability.
• M-TEER is safe and effective with favorable outcomes in experienced centers.

A 77-year-old woman presented to the emergency department with a 3-day history of chest pain. She was diagnosed with a delayed inferoposterior myocardial infarction and acute decompensated heart failure (New York Heart Association Class IV), complicated by respiratory failure requiring intubation. A murmur was noted on exam, and point of care ultrasound revealed significant mitral regurgitation (MR). Urgent cardiac catheterization with intraprocedural transesophageal echocardiogram (TEE) showed partial posteromedial papillary muscle rupture with P1/P2 prolapse and severe eccentric MR (Figure 1a and b). Coronary angiography demonstrated an occluded obtuse marginal branch (Figure 1c). Given prohibitive surgical risk, the heart team elected for concomitant percutaneous coronary intervention and mitral transcatheter edge to edge repair.

**Figure 1:**
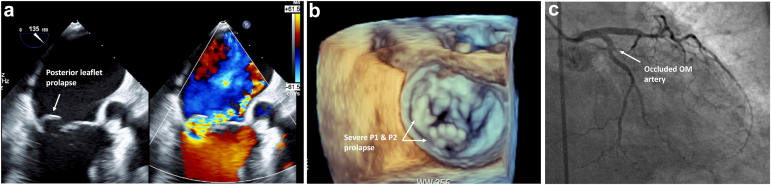
**Preprocedural imaging** **(a)** TEE with severe posterior leaflet prolapse and eccentric MR. **(b)** Three dimensional TEE rendering highlighting P1 and P2 scallop prolapse. **(c)** Coronary angiography demonstrating occluded OM artery. Abberviations: MR, mitral regurgitation; OM, obtuse marginal; TEE, transesophageal echocardiogram.

Right heart catheterization (RHC) showed elevated pulmonary capillary wedge pressure (mean 23 mmHg, v-wave 45 mmHg). Following RHC, IV heparin was given to achieve target activated clotting time, and obtuse marginal was stented with a 2.75 × 16 mm drug-eluting stent, restoring thrombolysis in myocardial infarction 3 flow (Figure 2a). An intra-aortic balloon pump was inserted post-percutaneous coronary intervention due to elevated mean arterial pressure (93 mmHg) with evidence of elevated systemic vascular resistance (1836 dynes/sec/cm^2^) and reduced cardiac index of 2.0 L/min/m^2^. The 7F sheath was exchanged for an 8.5 F Baylis sheath, which was advanced to the superior vena cava. Under fluoroscopic and TEE guidance, a coiled 0.035" radiofrequency wire was used to perform a posterior/mid-septal transseptal puncture. A 22F guide sheath was placed in the left atrium. A steerable Pascal ACE delivery system was introduced (Figure 2b), and a device was deployed at A2/P2 under 2D/3D TEE guidance (Figure 2c). A second device was placed at A1/P1 to address residual MR (Figure 2d and e). Final TEE showed a stable device position with trace MR (Supplemental Video 1). Repeat RHC showed improvement in pulmonary capillary wedge pressure to 15 mmHg, v-wave 20 mmHg. The patient was discharged to rehabilitation and continues to do well (Figure 2f and g).

**Figure 2:**
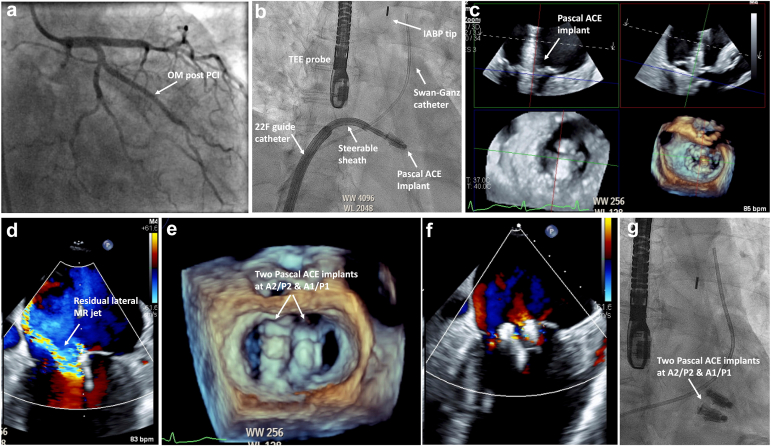
Intraprocedural imaging **(a)** Post-PCI. **(b)** LA access and Pascal ACE positioning. **(c)** Multiplanar reconstruction for TEE-guided implant positioning and leaflet capture. **(d)** Residual MR post A2/P2 implant. **(e)** 3D TEE post deployment of second implant. **(f)** Completion shot with 2 implants in appropriate position and minimal trace MR. **(g)** Completion fluoroscopic image. Abberviations: IABP, intra-aortic balloon pump; LA, left atrium; MR, mitral regurgitation; OM, obtuse marginal; TEE, transesophageal echocardiogram.

In select high-risk patients, mitral transcatheter edge to edge repair is a viable alternative to surgery for acute ischemic MR due to papillary muscle rupture. Several anatomical and procedural factors include limited atrial size affecting transseptal access, large flail gaps, risk of papillary muscle impingement, and hemodynamic instability.^1^ Given Pascal’s central spacer and broader paddles—features that offer an advantage in cases with a large flail gap—we elected to proceed with Pascal over MitraClip in this case. Nonetheless, when performed in experienced centers, this strategy can achieve effective hemodynamic stabilization.

## Consent Statement

Patient consent for publication was obtained by the authors.

## Funding

This research did not receive any specific grant from funding agencies in the public, commercial, or not-for-profit sectors.

## Disclosure Statement

A. Abbas has received research grants and consulting fees from 10.13039/100006520Edwards Lifesciences. I. Hanson is a proctor for 10.13039/100006520Edwards Lifesciences. The other authors had no conflicts to declare.

## Supplementary Material

Video 1 Final TEE showing trace residual MR. MR, mitral regurgitation; TEE, transesophageal echocardiogram.
